# Work-related accidents involving venomous animals in Brazil (2019 2023): associated factors and health inequalities among workers

**DOI:** 10.47626/1679-4435-2026-1510

**Published:** 2026-02-27

**Authors:** Samuel Santos Souza, Bruna Reis Santos, Jaqueline Santos Nascimento, Maria Luiza Santana da Costa, Thaylla Rodrigues Cerqueira

**Affiliations:** 1 Centro Universitário de Excelência (UNEX), Grupo de Estudo e Pesquisa em Saúde Ambiente e Trabalho (GEPSAT), Jequié, BA, Brazil; 2 Centro Universitário de Excelência (UNEX), Enfermagem, Grupo de Estudo e Pesquisa em Saúde Ambiente e Trabalho (GEPSAT), Jequié, BA, Brazil; 3 Centro Universitário de Excelência (UNEX), Biomedicina, Grupo de Estudo e Pesquisa em Saúde Ambiente e Trabalho (GEPSAT), Jequié, BA, Brazil; 4 Centro Universitário de Excelência (UNEX), Farmácia, Grupo de Estudo e Pesquisa em Saúde Ambiente e Trabalho (GEPSAT), Jequié, BA, Brazil

**Keywords:** occupational accidents registry, animals, poisonous, occupational health, healthcare disparities, socioeconomic factors., acidentes de trabalho, animais peçonhentos, saúde ocupacional, desigualdades de saúde, fatores socioeconômicos.

## Abstract

**Introduction:**

In Brazil, accidents involving venomous animals represent a significant health problem, affecting particularly vulnerable populations.

**Objectives:**

To analyze the factors associated with work-related accidents involving venomous animals in Brazil between 2019 and 2023.

**Methods:**

A cross-sectional study using secondary data from the Notifiable Diseases Information System, including sociodemographic variables, accident characteristics, and clinical manifestations. The analysis comprised absolute and relative frequencies, prevalence ratios (PR) with 95% confidence intervals, and robust Poisson regression for the multivariate model. Multicollinearity was assessed using the Variance Inflation Factor, and discriminatory ability was evaluated with the ROC curve.

**Results:**

Of the 1,442,464 reported cases of accidents involving venomous animals, 1,280,223 had valid data regarding work-relatedness; among these, 122,608 (9.58%) were classified as work-related. The mean age of victims was 36.5 years (SD = 21.0). Factors statistically associated with the outcome included: male sex (PR=2.66), black/brown race (PR=1.08), yellow/indigenous race (PR=1.24), illiteracy (PR=1.11), accidents involving snakes (PR=2.27), spiders (PR=1.19), and other animals (PR=1.18), as well as injuries to upper limbs (PR=1.23) and head (PR=1.12). The model showed an area under the ROC curve of 72.56% and a variance inflation factors average of 2.15, indicating good discriminatory capacity and absence of multicollinearity.

**Conclusions:**

Work-related accidents involving venomous animals predominantly affect vulnerable populations, reflecting social inequalities and gaps in worker health protection. The importance of preventive strategies expanded access to healthcare, and active surveillance in the territories-especially rural areas-is emphasized.

## Resumo

**Introdução:**

No Brasil, os acidentes envolvendo animais peçonhentos constituem um agravo de grande impacto para a saúde pública, afetando principalmente populações em situação de vulnerabilidade.

**Objetivos:**

Analisar os fatores associados aos acidentes de trabalho com animais peçonhentos no Brasil no período de 2019 a 2023.

**Métodos:**

Estudo transversal com dados secundários provenientes do Sistema de Informação de Agravos de Notificação, incluindo variáveis sociodemográficas, características do acidente e manifestações clínicas. Foram realizadas análises descritivas com frequências absolutas e relativas, estimativas de razão de prevalência (RP) com intervalos de confiança de 95% e regressão de Poisson com variância robusta para o modelo multivariado. A multicolinearidade foi avaliada por meio do fator de inflação da variância (FIV), e a capacidade discriminatória do modelo foi verificada pela análise da área sob a curva (AUC).

**Resultados:**

Entre 1.442.464 casos notificados de acidentes com animais peçonhentos, 1.280.223 apresentavam informações válidas quanto à relação com o trabalho, dos quais 122.608 (9,58%) foram classificados como relacionados ao trabalho. A média de idade das vítimas foi de 36,5 anos (desvio padrão = 21,0). Permaneceram estatisticamente associados ao desfecho: sexo masculino (RP = 2,66), raça preta/parda (RP = 1,08), raça amarela/indígena (RP = 1,24), escolaridade analfabeta (RP = 1,11), acidentes com serpentes (RP = 2,27), aranhas (RP = 1,19) e outros animais (RP = 1,18), além de acometimento em membros superiores (RP = 1,23) e cabeça (RP = 1,12). O modelo apresentou AUC de 72,56% e FIV médio de 2,15, indicando adequada capacidade discriminatória e ausência de multicolinearidade relevante.

**Conclusões:**

Os acidentes de trabalho com animais peçonhentos concentram-se em grupos socialmente vulneráveis, evidenciando desigualdades sociais e fragilidades nas ações de proteção à saúde do trabalhador. Destaca-se a necessidade de fortalecer estratégias preventivas, ampliar o acesso aos serviços de saúde e intensificar a vigilância ativa nos territórios, especialmente em áreas rurais.

## INTRODUCTION

Occupational accidents represent a significant public health issue in Brazil, affecting thousands of workers each year and generating direct impacts on the physical, mental, and economic well-being of the working population. In 2022, more than 612,000 occupational accidents were reported nationwide, with higher concentrations in the construction, agriculture, livestock, and urban service sectors, which are often characterized by informality and increased social vulnerability [1].

Venomous animals are those that possess venom-producing glands and specialized structures, such as fangs, stingers, or spines, that allow the inoculation of toxins. The main representatives of this group include snakes, spiders, bees, scorpions, and caterpillars, etc. [2].

Due to its continental dimensions, the presence of tropical and subtropical zones, and high biodiversity associated with a predominantly tropical climate, Brazil offers favorable conditions for the wide distribution of venomous species such as snakes, spiders, and scorpions. Bites and stings may result in clinically relevant and potentially severe manifestations, making preventive measures and timely access to health care essential to avoid complications and reduce risks to the population [3].

In this context, accidents involving venomous animals represent a major public health concern, particularly affecting socially vulnerable populations. Evidence indicates that the main victims are young adult males, especially rural workers who are routinely exposed to high-risk environments. The occurrence of these accidents tends to be higher in rural and peri-urban areas, where these animals are more common and access to health services may be limited [4].

Among work-related environmental hazards, accidents involving venomous animals stand out due to their high frequency, potential clinical severity, and wide territorial distribution. Data from the Notifiable Diseases Information System (SINAN) indicate that, in 2023 alone, more than 300,000 cases were reported in Brazil, placing this type of event among the leading causes of morbidity associated with environmental factors [5]. Despite their relevance, studies specifically investigating the direct relationship between venomous animal accidents and the occupational context remain limited.

Therefore, the present study aims to analyze the factors associated with occupational accidents involving venomous animals in Brazil from 2019 to 2023.

## METHODS

### STUDY DESIGN AND DATA SOURCE

This is an exploratory cross-sectional epidemiological study based on the analysis of notification records of accidents involving venomous animals. Data were obtained electronically from the Health Information platform of the Notifiable Diseases Information System (SINAN), maintained by the Department of Informatics of the Unified Health System (SUS), covering the period from 2019 to 2023.

The following variables were included: i) sociodemographic data: age (< 18 years; 18-30 years; 31-59 years; 60 years or more), sex (male, female), race/ethnicity (White, Black/Brown, Asian/Indigenous), and education level (illiterate, elementary school, high school, higher education); ii) accident characteristics: type of venomous animal (scorpion, snake, spider, others), body region affected (trunk, upper limbs, head, lower limbs), receipt of medical care (yes, no), case outcome (recovery, death), and time elapsed between the accident and medical care, in hours (up to 1 h; 1-3 h; 3-6 h; more than 6 h); iii) clinical characteristics: local manifestations (yes, no), systemic manifestations (yes, no), clotting time (normal, abnormal), case classification (mild, moderate, severe), local complications (yes, no), and systemic complications (yes, no).

The primary outcome of the study was whether the accident was work-related, categorized as “yes” or “no.”

### STATISTICAL ANALYSIS

A descriptive characterization of individuals involved in accidents with venomous animals was initially performed. Categorical variables were presented as absolute and relative frequencies, while age was described using mean and standard deviation (SD).

Bivariate analysis was then conducted to investigate the association between sociodemographic variables, accident characteristics, and clinical variables with the outcome. The measure of occurrence used was prevalence (P%), calculated as the ratio between the number of work-related accidents and the total number of accidents involving venomous animals for each variable analyzed. The measure of association adopted was the prevalence ratio (PR), with corresponding 95% CIs.

To construct the final model, Poisson regression with robust variance was used, which is appropriate for cross-sectional studies with a binary outcome, to estimate adjusted PRs. Variables with p < 0.20 in the bivariate analysis were initially included in the multivariable model. Final selection followed a backward strategy, with retention criteria based on a Wald test value ≤ 0.05 and the lowest Akaike information criterion.

Model fit was assessed by analyzing the area under the receiver operating characteristic curve (AUC). The presence of multicollinearity among independent variables was evaluated using the variance inflation factor (VIF). All analyses were performed using Stata statistical software, version 12.

### ETHICAL CONSIDERATIONS

As this study was based on publicly available secondary data, submission to a Research Ethics Committee was not required. The principles established by Resolution no. 466/2012 of the Brazilian National Health Council were followed. The data used are anonymized, contain no personal identifiers, and are publicly accessible without the need for formal authorization.

## RESULTS

Of the 1,442,464 reported cases of accidents involving venomous animals, 1,280,223 had valid information regarding their relationship to work. Among these, 122,608 (9.58%) were classified as work-related. The mean age of the affected individuals was 36.5 years (SD = 21.0).

The analysis of Table 1 demonstrates statistically significant associations between sociodemographic characteristics and the occurrence of occupational accidents involving venomous animals. Regarding sex, male workers showed a substantially higher P% (14.18%) compared with female workers (3.95%), with a PR of 3.59 (95% CI, 3.54-3.65), indicating that men experience approximately 3.6 times more occurrences of this type of accident.

**Table 1:** Prevalence of work-related accidents involving venomous animals according to sociodemographic characteristics. Jequié, Bahia, 2026

Variables	n	P%	PR	95% CI	p-value
Sex					
Female	22,699	3.95	1.00		
Male	99,902	14.18	3.59	3.54-3.65	0.001
Age (years)					
60 or more	15,025	7.25	1.00		
31-59	69,632	13.22	1.82	1.79-1.85	0.001
18-30	32,176	11.97	1.65	1.62-1.68	0.001
Under 18	2,944	4.39	0.60	0.58-0.62	0.001
Race					
White	40,889	9.38	1.00		
Black/Brown	71,965	9.36	1.06	1.05-1.07	0.001
Asian/Indigenous	4,047	16.15	1.72	1.67-1.77	0.001
Education level					
Higher education	5,587	9.57	1.00		
Illiterate	41,877	13.01	1.35	1.32-1.39	0.001
High school	21,008	9.76	1.01	0.99-1.04	0.170
Elementary school	18,655	11.68	1.21	1.18-1.25	0.001

P% = prevalence; PR = prevalence ratio.

With respect to age group, individuals aged 31-59 years presented the highest proportion of work-related accidents (PR, 1.82; 95% CI, 1.79-1.85) compared with those aged 60 years or older. Workers aged 18-30 years also showed a higher occurrence (PR = 1.65), whereas individuals younger than 18 years had a significantly lower proportion (PR, 0.60; 95% CI, 0.58-0.62).

In terms of race/ethnicity, individuals self-identified as Asian/Indigenous showed the highest PR (1.72; 95% CI, 1.67-1.77), followed by Black/Brown individuals (PR, 1.06; 95% CI, 1.05-1.07), when compared with the reference group composed of White workers.

Regarding education level, a higher occurrence was observed among illiterate workers, with a P% of 13.01% and a PR of 1.35 (95% CI, 1.32-1.39) compared with those with higher education. Workers with elementary education also showed a higher proportion of accidents (PR, 1.21; 95% CI, 1.18-1.25), whereas those with high school education did not demonstrate a statistically significant difference compared with the reference group.

According to Table 2, regarding the type of causative animal, accidents involving snakes showed a higher likelihood of being work-related (PR, 3.49; 95% CI, 3.45-3.54) compared with those caused by scorpions. Accidents involving spiders and other venomous animals were also more strongly associated with occupational exposure than the reference group, both with a PR of 1.47 (95% CI, 1.44-1.49).

**Table 2:** Prevalence of work-related accidents involving venomous animals according to accident characteristics. Jequié, Bahia, 2026

Variables	n	P%	PR	95% CI	p-value
Type of animal					
Scorpion	53,244	6.83	1.00		
Snake	33,662	23.88	3.49	3.45-3.54	0.001
Spider	16,009	10.05	1.47	1.44-1.49	0.001
Others	18,394	10.04	1.47	1.44-1.49	0.001
Body region affected					
Trunk	5,626	7.75	1.00		
Upper limbs	62,179	11.42	1.47	1.43-1.51	0.001
Head	9,987	11.66	1.50	1.45-1.55	0.001
Lower limbs	43,534	8.02	1.03	1.00-1.06	0.001
Medical care received					
Yes	117,687	9.81	1.00		
No	3,010	5.21	0.53	0.51-0.55	0.001
Case outcome					
Recovered	113,634	9.33	1.00		
Death	346	20.27	2.17	1.97-2.38	0.001
Time between accident and medical care					
Up to 1 h	56.855	8.41	1.00		
1-3 h	33.365	11.93	1.41	1.40-1.43	0.001
3-6 h	12.707	14.40	1.71	1.68-1.74	0.001
More than 6 h	14.760	9.46	1.12	1.10-1.14	0.001

P% = prevalence; PR = prevalence ratio.

Concerning the body region affected, accidents involving the upper limbs showed a PR of 1.47 (95% CI, 1.44-1.49), while those affecting the head showed a PR of 1.50 (95% CI, 1.45-1.55). Accidents involving the lower limbs also demonstrated a higher association (PR, 1.03; 95% CI, 1.00-1.06). Overall, these findings indicate a higher occurrence of occupational accidents compared with those affecting the trunk.

With respect to medical care, cases that did not receive assistance showed a PR of 0.53 (95% CI, 0.51-0.55), which indicates a lower association with work-related accidents.

Regarding case outcomes, events that resulted in death showed a stronger association with the occupational context (PR, 2.17; 95% CI, 1.97-2.38) compared with cases that progressed to recovery.

Finally, concerning the time elapsed between the accident and medical care, a positive association with the outcome was observed for care provided between 1 and 3 hours (PR, 1.41; 95% CI, 1.40-1.43), between 3 and 6 hours (PR, 1.71; 95% CI, 1.68-1.74), and after more than 6 hours (PR, 1.12; 95% CI, 1.10-1.14), compared with cases that received care within 1 hour after the accident.

Table 3 shows that, regarding systemic manifestations, workers who presented these conditions showed a stronger association with work-related accidents (PR, 1.72; 95% CI, 1.69-1.75) compared with those without such manifestations.

**Table 3:** Prevalence of work-related accidents involving venomous animals according to clinical characteristics. Jequié, Bahia, 2026

Variables	n	P%	RP	IC 95%	Valor p
Local manifestations					
No	5,606	9.44	1.00		
Yes	116,062	9.60	1.01	0.99-1.04	0.2019
Systemic manifestations					
No	107,417	9.19	1.00		
Yes	11,831	15.88	1.72	1.69-1.75	0.001
Clotting time					
Normal	19,657	13.44	1.00		
Altered	8,377	17.14	1.27	1.24-1.30	0.001
Case classification					
Mild	93,862	8.52	1.00		
Moderated	21,999	16.69	1.95	1.93-1.98	0.001
Severe	3,646	18.97	2.22	2.16-2.29	0.001
Local complications					
No	113,021	9.30	1.00		
Yes	2,893	20.18	2.16	2.09-2.24	0.001
Systemic complications					
No	113,576	9.32	1.00		
Yes	1,005	22.37	2.40	2.27-2.53	0.001

P% = prevalence; PR = prevalence ratio.

With respect to clotting time, individuals with altered results demonstrated a higher association (PR, 1.27; 95% CI, 1.24-1.30) compared with those whose clotting time was within the normal range.

Case classification revealed a progressive increase in association according to the severity of the accident in the occupational setting. Moderate cases showed a PR of 1.95 (95% CI, 1.93-1.98), and severe cases a PR of 2.22 (95% CI, 2.16-2.29), both compared with cases classified as mild.

Regarding the presence of local complications, cases presenting this type of complication showed a higher association with work-related accidents (PR, 2.16; 95% CI, 2.09-2.24) compared with those without complications.

Finally, individuals with systemic complications demonstrated an even stronger association (PR, 2.40; 95% CI, 2.27-2.53) compared with cases without this type of complication (P% = 9.32%).

In the final multivariable Poisson regression model presented in Table 4, the variables that remained statistically associated with work-related accidents involving venomous animals were male sex (PR, 2.66; 95% CI, 2.56-2.76), Black/Brown race (PR, 1.08; 95% CI, 1.05-1.11), Asian/Indigenous race (PR, 1.24; 95% CI, 1.16-1.32), and illiteracy (PR, 1.11; 95% CI, 1.06-1.17).

**Table 4:** Final multivariable regression model for work-related accidents involving venomous animals. Jequié, Bahia, 2026

Variables	PR	95% CI	p-value
Sex			
Male	2.66	2.56-2.76	0.001
Race			
Black/Brown	1.08	1.05-1.11	0.001
Asian/Indigenous	1.24	1.16-1.17	0.001
Education level			
Illiterate	1.11	1.06-1.17	0.001
High school completed	0.99	0.94-1.05	0.730
Elementary school completed	1.05	0.99-1.11	0.113
Type of animal			
Snake	2.27	2.18-2.35	0.001
Spider	1.19	1.13-1.26	0.001
Others	1.18	1.11-1.24	0.001
Body region affected			
Upper limbs	1.23	1.14-1.33	0.001
Head	1.12	1.02-1.23	0.023
Lower limbs	0.82	0.75-0.88	0.001
Time to care			
1-3 h	1.10	1.06-1.13	0.001
3-6 h	1.13	1.09-1.18	0.001
More than 6 h	0.99	0.95-1.03	0.622
Systemic manifestations			
Yes	1.17	1.13-1.21	0.001
Clotting time			
Altered	0.97	0.94-1.00	0.051
Case classification			
Moderate	1.19	1.15-1.23	0.001
Severe	1.13	1.07-1.20	0.001
Local complications			
Yes	1.24	1.18-1.31	0.001

PR = prevalence ratio.

Regarding the type of animal, accidents involving snakes showed a PR 2.27 times higher (95% CI, 2.18-2.35), followed by spiders (PR, 1.19; 95% CI, 1.13-1.26) and other animals (PR, 1.18; 95% CI, 1.11-1.24), compared with accidents caused by scorpions.

Concerning the body region affected, accidents involving the upper limbs (PR, 1.23; 95% CI, 1.14-1.33) and head (PR, 1.12; 95% CI, 1.02-1.23) showed a positive association with work-related accidents, whereas injuries to the lower limbs showed a negative association (PR, 0.82; 95% CI, 0.75-0.88), compared with injuries to the trunk.

The time between the accident and medical care was also relevant. Intervals of 1-3 hours (PR, 1.10; 95% CI, 1.06-1.13) and 3-6 hours (PR, 1.13; 95% CI, 1.09-1.18) were associated with a higher prevalence of work-related accidents.

In addition, the presence of systemic manifestations was associated with the outcome (PR, 1.17; 95% CI, 1.13-1.21), as were moderate (PR, 1.19; 95% CI, 1.15-1.23) and severe case classifications (PR, 1.13; 95% CI, 1.07-1.20). The occurrence of local complications also increased the PR (1.24; 95% CI, 1.18-1.31), indicating greater severity among work-related cases.

The model showed an AUC of 72.56%, indicating good discriminative ability. VIF analysis yielded a mean value of 2.15, with all variables below the cutoff point of 10, indicating the absence of significant multicollinearity.

## DISCUSSION

This epidemiological, descriptive, and analytical study investigated factors associated with work-related accidents involving venomous animals in Brazil, with emphasis on sociodemographic characteristics, accident-related factors, and clinical manifestations. The findings highlight important social inequalities related to the profile of affected workers, the severity of events, and the response of health services. The results are discussed according to thematic categories, in light of the scientific literature and the principles of public health.

Men showed an approximately 3.6-fold higher risk of occupational accidents involving venomous animals compared with women. This result reflects the sexual division of labor, in which men are more frequently engaged in activities with greater exposure to risks, such as agriculture, construction, and waste handling. These findings are consistent with previous studies on occupational accidents, particularly in rural settings [2,6].

Regarding age, adults between 31 and 59 years were the most affected, corresponding to the most economically active segment of the population. The overlap between working age and a higher occurrence of occupational accidents has been identified as a risk factor in other epidemiological investigations, reinforcing the impact of these events on the workforce and productivity [4,7].

Data also showed a higher prevalence among workers self-identified as Asian/Indigenous and Black/Brown. This inequality should be examined in the context of the social determinants of health. Racialized populations often face greater socioeconomic vulnerability, are more frequently employed in informal and high-risk occupations, and experience the effects of structural racism, which limits access to labor rights and health protection [8,9].

Workers who were illiterate or had only elementary education also showed a higher prevalence of accidents. Lower educational attainment is associated with employment in informal and higher-risk occupations, often with limited provision of personal protective equipment (PPE) and reduced access to training. This scenario increases the likelihood of accidents and makes it more difficult to adopt preventive practices. In addition, limited access to information on protective measures may contribute to increased exposure to risk situations [10,11].

The results indicated that accidents involving snakes were approximately 3.5 times more likely to be work-related compared with those involving scorpions. This finding may be explained by the greater presence of snakes in rural areas, where outdoor labor activities such as farming, land clearing, and vegetation management are common. This context increases direct contact with these animals and the likelihood of accidents during working hours [12,13].

Scorpions, although responsible for the majority of venomous animal accidents in Brazil and widely distributed in urban environments, showed the lowest association with work-related accidents in this study. This result may be related to the fact that such incidents occur more frequently in domestic settings rather than in occupational contexts [5,14].

Regarding the body region affected, the upper limbs and head were the most frequently involved in occupational accidents with venomous animals, with PRs of 1.47 and 1.50, respectively, compared with the trunk. This pattern may be explained by the frequent use of the hands and the exposure of the head during work activities, particularly in occupations involving direct contact with soil, debris, or vegetation, such as agriculture, construction, and waste collection. Direct exposure and the absence or improper use of PPE, such as gloves and helmets, likely contribute to increased risk. These findings are consistent with a study conducted in the Federal District, which also identified the upper limbs as the most affected areas in occupational accidents involving venomous animals and highlighted gaps in preventive practices and safety culture among exposed workers [15,16].

With regard to medical care, cases in which the victim did not receive assistance showed a lower prevalence of association with work-related accidents. This may indicate less severe incidents or underreporting, particularly when the event is not formally recognized as occupational. Conversely, cases with a time to care exceeding three hours showed a higher prevalence of association with work-related accidents, suggesting that workers, especially in rural areas, face greater barriers in accessing health services. Geographic distance and infrastructure limitations may delay care, which is particularly concerning given that timely treatment is a key determinant of prognosis and clinical outcomes. Previous studies have shown that delayed access to health services is associated with worse outcomes and increased severity in occupational accidents [17,18].

Data analysis also indicated that occupational accidents involving venomous animals tend to present greater severity and impact, particularly when resulting in fatal outcomes. Cases that progressed to death showed a 2.17-fold higher association with the occupational context, highlighting the seriousness of these events, especially those involving snakes. The venom of these animals can cause severe complications within a short time frame, and delays in emergency care represent an important aggravating factor [19]. According to Alvarenga et al. [2], it is essential to improve preventive strategies, working conditions, and timely access to medical care for rural workers in order to reduce the occurrence of injuries and deaths associated with these events.

Regarding clinical manifestations, individuals who presented systemic manifestations, altered clotting time, complications, and greater case severity showed stronger associations with work-related accidents involving venomous animals compared with non-occupational cases. These findings suggest that occupational events tend, on average, to be more severe [13]. Such data reinforce the importance of primary prevention in the workplace, including proper use of PPE, continuous worker training, and strengthening active surveillance actions, as recommended by occupational health surveillance within the SUS framework [20].

Some limitations of this study should be acknowledged. The analysis was based on secondary data from SINAN, which may be subject to incomplete reporting, inconsistencies, and underreporting, particularly in hard-to-reach areas and in cases considered less severe. Previous studies, such as that by Fiszon & Bochner [21], have shown that although essential for epidemiological surveillance, the system still has limitations in fully capturing cases of accidents involving venomous animals. This may affect the accurate estimation of the problem’s magnitude and influence the development of prevention and response strategies. Despite these limitations, the findings contribute to expanding knowledge on occupational accidents involving venomous animals and reinforce the need to improve data quality and timely access to health services.

Occupational accidents involving venomous animals result in relevant clinical and social consequences. In this context, the importance of occupational health surveillance actions is reinforced, in accordance with SUS guidelines and public health principles. The implementation of health promotion strategies in workplace settings, encouragement of proper PPE use, intersectoral collaboration, and integration across different levels of care, including Worker Health Reference Centers, are essential for early detection of cases and for improving the quality of care [8].

## CONCLUSIONS

This study showed that factors such as sex, race/ethnicity, education level, and access to health services significantly influence the risk of occupational accidents involving venomous animals. Men were more likely to experience this type of accident, possibly due to their greater participation in occupations with high exposure to risk. Racialized populations, including Black, Brown, Indigenous, and Asian workers, also demonstrated greater vulnerability, reflecting social inequalities, higher representation in informal employment, and the effects of structural racism.

In addition, low educational attainment and difficulties in accessing medical care, especially in rural areas, contribute to worse outcomes by limiting both prevention and timely, adequate treatment.

The implementation of preventive measures is essential, including the proper use of PPE, the provision of training, and the development of educational campaigns aimed at reducing occupational exposure. Strengthening public policies targeted at the most vulnerable groups is also crucial, with a focus on social inclusion, expanded access to health services, and the reduction of racial inequalities. Studies such as this provide important evidence to support the development of more effective strategies, contributing to the promotion of safer, more equitable, and more inclusive work environments.
